# Revealing the morphological architecture of a shape memory polyurethane by simulation

**DOI:** 10.1038/srep29180

**Published:** 2016-07-04

**Authors:** Jinlian Hu, Cuili Zhang, Fenglong Ji, Xun Li, Jianping Han, You Wu

**Affiliations:** 1 Institute of Textiles and Clothing, the Hong Kong Polytechnic University, Hung Hom, Kowloon, Hong Kong, China; The Hong Kong Polytechnic University Shenzhen Base, Shenzhen, China; 2 School of Textiles and Clothing, Wuyi University, Jiangmen, Guangdong, 529020, China; 3 Department Applied Mathematics, the Hong Kong Polytechnic University, Hung Hom, Kowloon, Hong Kong, China

## Abstract

The lack of specific knowledge of the network structure in shape memory polymers (SMPs) has prevented us from gaining an in-depth understanding of their mechanisms and limited the potential for materials innovation. This paper firstly reveals the unit-cell nanoscale morphological architecture of SMPs by simulation. The phase separated architecture of a segmented shape memory polyurethane (SMPU) with a 30 wt% hard segment content (HSC, 4,4’-diphenylmethane diisocyanate (MDI) and 1,4-butanediol (BDO)) showing good shape memory properties was investigated by dissipative particle dynamics (DPD) simulations. A linked-spherical netpoint-frame phase of MDI, a matrix-switch phase of polycaprolactone (PCL) and a connected-spider-like interphase for BDO were obtained for this SMPU. The BDO interphase can reinforce the MDI network. Based on these simulation results, a three-dimensional (3D) overall morphological architectural model of the SMPU can be established. This theoretical study has verified, enriched and integrated two existing schematic models: one being the morphological model deduced from experiments and the other the frame model for SMPs reported before. It can serve as a theoretical guide for smart polymeric materials design. This method for the simulation of polymer structure at the nanoscale can be extended to many areas such as photonic crystals where nanoscale self-assembly plays a vital role.

Smart materials have a unique ability to respond to stimuli^1^,^2^, and perform as both sensors and actuators as well as general functional components. They are smart due to their exceptional or delicate structures. During the past two decades, much research has been dedicated to develop SMPs because of their scientific and technological significance^3^,^4^,^5^,^6^,^7^,^8^. The SMPs generally have a structure of soft and hard segments and an optimal combination of netpoints and switches. Understanding structural details of shape memory polymers is extremely important for achieving specific properties and designing innovative materials. An SMP recovers its initial shape in response to certain external stimuli, such as heat^7^, light^9^, electricity^10^, magnetism^11^, water^12^ or solvent^13^, pressure^14^ etc. In particular, the shape-memory effect of segmented polyurethane (PU) block copolymers has been researched extensively because of their superior material properties, and thermal-responsive SMPUs are one of the most widely explored in materials^15^. Generally, a thermal-responsive SMPU consists of two components, hard segments (urethane or urea) controlling the permanent shape, and soft segment (mainly polyether or polyester) fixing the temporary shape at temperatures below the transition level (T_trans_), which is either the glass transition (T_g_) or melting temperature (T_m_) of the switching segments.

Self-assembly of block copolymers can yield versatile hybrid materials with diverse applications^16^, such as wire networks^17^,^18^, lithographic templates^19^ , solid electrolytes^20^, and photonic crystals (PCs)^14^,^21^. The self-assembly of PUs can produce a wide range of different morphologies. Spheres (“islands”)^22^,^23^, cylinders^22^, and lamellar-like morphologies^24^, straight whiskers and fibre-bundle-like morphologies^25^ have been reported in literature, and are similar to morphologies known for diblock copolymers^26^. Typically the microstructural size scale is on the order of 10 nm^27^, and one of 30 nm was found for the hard segment domain in a 51 wt % hard segment PU^28^. In our previous work^29^, the presence of an isolated hard segment domain (see Fig. 1d) was deduced from a combination of differential scanning calorimetry (DSC) (see Fig. 1b), small angle X-ray scattering (SAXS) (see Fig. 1c), and thermomechanical analysis (TMA) (see Fig. 1a) for a MDI-SMPU with 30 wt% hard segment content showing good shape memory properties. However, no details or visual phase structures were presented for this material.

Miller *et al.*^30^ used a Monte Carlo (MC) simulation developed for exploring premature phase-separation during reaction of polyurethane block copolymers. Tao *et al.*^31^ applied a combination of a molecular dynamics (MD) simulation and the MC method to calculate the phase diagram and degree of phase separation of polyurethanes. Raghu *et al.*^32^ performed MD simulation to produce X-ray diffraction (XRD) patterns for determining the phase morphology of the PUs. The dissipative particle dynamics (DPD) method developed by Hoogerbrugge and Koelman^33^,^34^ is a mesoscopic simulation technique for complex fluids that can study systems over larger length and longer time scales than classical MC and MD simulations. It has been successfully applied to polymeric systems by introducing bead-spring type models^35^,^36^,^37^ and it is particularly suitable to investigate the microphase separation and rheological properties of block copolymers and polymer blends^38^,^39^. Sami *et al.* used diblock structure, namely, without chain extender, investigated the influence of diisocyanate symmetry and nature of the hydrogen bonding between different hard segments on the morphology development of two-segmented polyurethanes and polyureas first by quantum mechanical calculations and then by DPD simulations. They found that long-range connectivity of hydrogen bonds between urethane and urea groups in PPDI based segmented copolymers can form microphase separated morphologies, but the kinked MPDI based copolymers do not display well-defined microphase separation. Their polyurethanes have simplified structures and results are generally qualitative, which are not intended for shape memory polyurethane^40^.

In our previous work, a generalized 3D frame model of a SMP was proposed based on the development of molecular mechanisms^41^, see Fig. 1e. In this model, the SMPs contain both switch units and net-points. In literature, several other researchers attempted to propose similar shape memory polymer models for illustrating the mechanism of shape memory effect. Most models contain both switch units and net-points^3^,^42^,^43^ without accurate structural details. Instead they are mainly based on qualitative schematics. Usually, the quantity of hard segment phase is very small and described as net-points, which may demonstrate that the shape memory materials should have low hard segment content. Furthermore, a number of papers have studied the morphologies of polyurethanes in both experiments and simulations^22^,^23^,^24^,^25^,^27^,^28^,^29^,^30^,^31^,^32^,^40^. There is still a question, however, of how accurate these morphological and frame models are for a specific polymer and as a whole. According to our knowledge, no attempt was made to give detailed phase information for the shape memory polymer models specifically and none of them have focused on revealing the 3D unit-cell architecture of shape memory polymer models in terms of phase separation using theoretical methods. Instead they are mainly based on qualitative schematics.

Practically, due to the structural complexity, we found that it is easy to compute a large amount of data from the DPD simulations of a shape memory polymer, but normally very difficult to interpret: What is the meaning of all individual figures? Is there any stable pattern in all computed figures? Is this pattern reasonable? Why is this pattern like this? In addition, how to present the pattern if existing? What is the exact size of this pattern? What is the relationship between the simulated structural data and shape memory effects? In present study, we will attempt to provide a 3D unit-cell model for a SMPU meeting the above requirements in the present work. Specifically, in this paper, the morphology, namely phase separation and combination of the SMPU in two dimension and architectures in three dimensions will be simulated by the DPD method. The results will be associated to the morphological and frame models previously proposed. In this present study, the shape memory polymer structural model will be presented with phase domain details taking a SMPU with 30 wt% hard segment content as an example. A more accurate architectural model for SMPs will then be put forward, which will be a good guidance for the design of smart structures of functional materials in future.

## Results

For DPD simulations, several polymer-segments are systematically coarse-gained into a single simulation bead based on the molar volume of the monomers. Different beads are assumed to have equal volume, and the interaction parameters between beads are estimated from Flory-Huggins χ parameters obtained from the solubility parameters. The architectures of each component MDI, PCL and BDO are shown in Fig. 2. To better show the relative position of the phase domains of different components, the combination of phase domains for two components is given in Fig. 3. The overall phase structures of this SMPU with 30 wt% HSC are shown in Fig. 4.

### One Phase Architecture of SMPU

From the molecular structure of SMPU shown in Fig. 5, it is found that the MDI phase plays a key role in controlling the phase separation and forming the structural framework of SMPU. Thus, the architecture of MDI phase is presented firstly as shown in the left part of Fig. 2.

The MDI architecture shows that the MDI phase builds an integrated net-point framework with eight connected spherical domains in a unit-cell, similar phase structure was found in Mologin’s previous study^42^, who used the cellular-automaton-based simulation to study the structural organization of water-containing nafion. From the profile of the MDI in three axial surface planes, we can clearly find the details of the connections between the spherical phases. In the Y-Z plane, only the vertical and horizontal connections appear, while narrow connections occur between two diagonal spheres at 45 ° in Z-X plane, and much stronger connections are found between two diagonal spheres at around 135 ° in Y-X plane. Finally, a schematic drawing is shown with the essential features of the MDI architecture. It is interesting to see that it is similar to the net-points in our previous proposed shape memory frame model^41^, but with more detail such as the exact diagonal connections. Also in our previous report^29^, an isolated hard segment domain was deduced for the SMPU with 30 wt% HSC based on the data from several characteration methods, namely, TMA, SAXS and DSC. From the 3D structure of the MDI, we can see that the connections between spherical domains are relatively narrow compared to the size of the spheres, so our simulation results agree well with our experiments^29^.

A soft segment is an essential component for the shape memory polymers, but very little attention has been paid to its phase information. In this section, the analysis of its phase structures will be shown, see the middle part of Fig. 2. For SMPU with 30 wt% HSC, the PCL (soft segment) has its own phase, and linked perforated lamella phases or crossing cylinders appear, just like 4 × 4 × 8 cylinders square crossing in Y-Z, Z-X, and Y-X plane, respectively. From the profile structures, we can find that the PCL phase is connected as a whole without any gaps in the Y-Z plane, while a very small gap appears in the middle of four crossing cylinders in the Z-X plane, and a bigger hole is found in the middle of four crossing cylinders in the Y-X plane. The PCL takes up most of the space of the SMP for the particular hard segment content.

The right part of Fig. 2 shows the phase structures of another component of the hard segment – BDO. The shape of phase domain of BDO is not regular and not easy to describe, and shows different images in three planes: the BDO phase in the Y-Z plane looks like a linked-winter-hat in the Z direction, while in the Z-X plane, it appears as a linked-spider in the Z direction, and an isolated bottle in the Y-X plane. The schematic drawing shows the connections in the Z direction of the BDO phase, but separated in both the Y and X directions.

### Two Phase Architecture of SMPU

When the combination of the hard segments including MDI and BDO is shown, the phase morphologies of MDI and BDO seem to be unmixable in the SMPU as shown in the left part of Fig. 3 in contrary to the existing common belief. The phase structure of the hard segment is actually dominated by the MDI phase, which contributes to the basic framework of the polymer structure while the BDO phase is dependent on the MDI phase. The BDO phase is attached to the MDI phase in the unfilled regions and supports the formation of a stronger network. The schematic drawing clearly presents the connections between MDI phase and BDO phase.

To show the relative position of the phase domains between the PCL and MDI, we present combined phase structures of PCL and MDI in the middle part of Fig. 3. From the 3D phase architecture, we can see that the MDI forms a spherical net inlaid in the PCL bulk or PCL matrix. It is clear that, there are some gaps between the domains of PCL and MDI in different directions, which may refer to the phase domain of BDO. The schematic drawing shows the details of connected MDI domain inlaid in the PCL matrix, which have different connections of MDI spherical phase domain among the PCL phase domain.

The right part of Fig. 3 shows the combined phase structure of PCL and BDO. We can find that, the PCL phase takes up the most space of the material and the BDO surrounds the netpoints of the PCL phase domain which will be connected to the MDI phase as shown in the middle of this figure, indicating that the BDO phase acts as the interphase between PCL and MDI domains. The schematic drawing shows the attachment of the BDO phase domain to the PCL phase domain.

### Overall Morphological Architecture of SMPU

The overall morphological architecture of SMPUs with a hard-segment content of 30 wt% is presented in Fig. 4, which shows that the three components of this SMPU are immiscible with each other. The MDI phase forms the netpoint-frame while BDO segment surround the PCL and MDI phases and strengthens the framework. The hard segment phases (MDI and BDO together) are inlaid in the matrix of soft segment PCL. The PCL acts as the switch in the form of a matrix embracing both the MDI and BDO phases. The schematic drawing shows the domains of MDI and BDO fill in the soft segment matrix and the BDO phase domain strengthens the framework of net-points.

To better describe the phase domains of hard segment in the soft segment, we calculate the size of the MDI domains by measuring the size of the phase morphologies in Fig. 4. The MDI domain is in the scale of 14.5–16.5 nm in three directions, in good agreement with the literature^27^, while the size of the linkages between MDI spheres are in the range of 1.1–1.3 nm. Our calculated domain sizes are much larger than that determined from our previous SAXS results^29^, if we consider both interdomain spacings and domain size of the crystal as the phase size in literature^29^, our simulation results are also compared well with the experimental results^29^, which may arise from different definitions of domain sizes for two methods.

## Discussions

**Figure 1:**
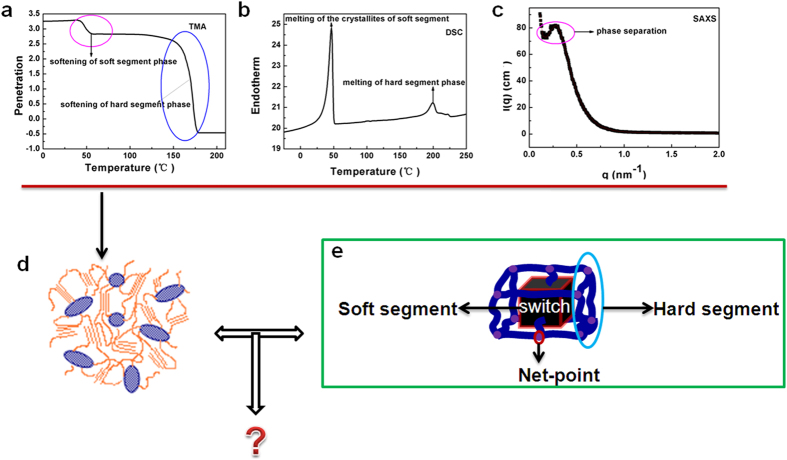
Structural models of shape memory polymers^29^,^41^. (**a**) TMA curve. (**b**) DSC curve. (**c**) SAXS curve. (**d**) Morphological model of SMP. (**e**) Frame model with switch-net-points of SMP.

**Figure 2:**
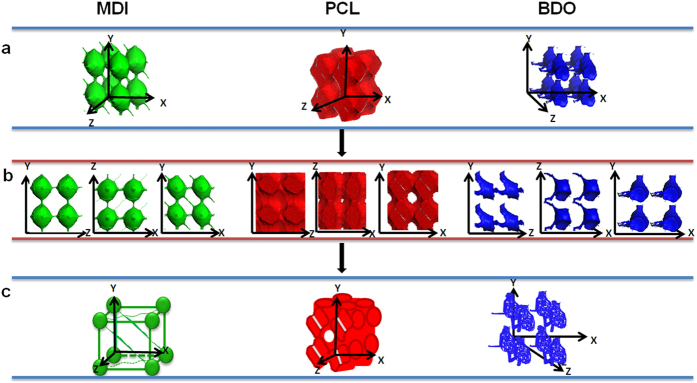
Architecture of each component in SMPU (green represents MDI, red PCL, blue BDO). (**a**) 3D architecture. (**b**) 2D morphologies in the different orthogonal planes. (**c**) 3D schematic architectural model for each component.

**Figure 3:**
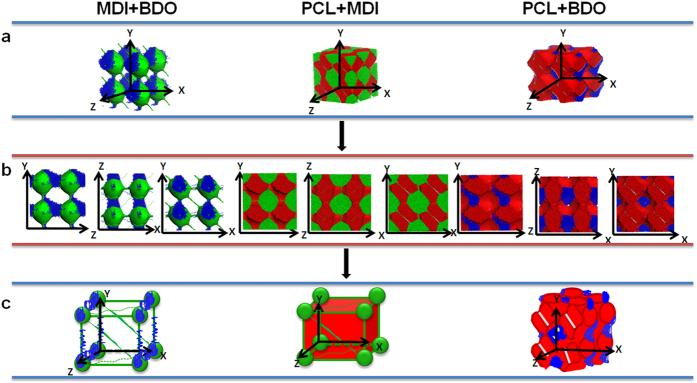
Architecture of the collective mode of two components for SMPU. (**a**) 3D architecture of SMPU. (**b**) 2D morphologies. (**c**) Schematic 3D architecture models of SMPU.

**Figure 4:**
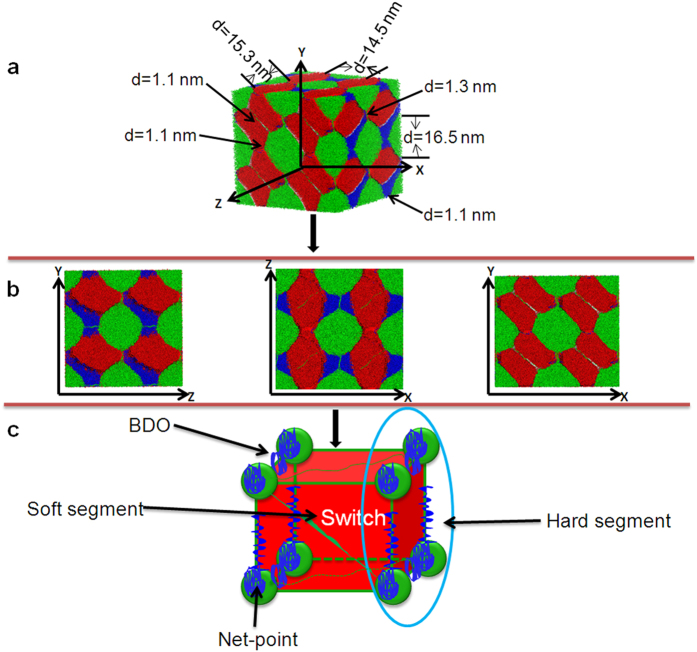
Overall morphological architecture of a SMPU. (**a**) Simulated architecture with domain size. (**b**) Simulated morphologies at three directions. (**c**) Schematic architectural model of the SMPU.

Shape memory polymers have unique microstructures which lead to their smart behavior, namely remembering their shapes upon an external stimulus. Thus understanding the details of this microstructure is extremely important in effectively designing and fabricating such materials. The SMPU is one of the most important polymers offering wide range of shape memory properties with high flexibility in molecular and morphological design. It is our objective of the present study to verify two schematic models: namely, morphological model (Fig. 1d) and netpoint-switch network model (Fig. 1e) of a shape memory polymer through a typical example of polyurethane by a DPD simulation. As reported by Ji *et al.*^29^, shape memory effects of segmented polyurethanes change with hard segment contents (HSCs) where intermediate HSCs of 25 wt%–30 wt% lead to both good mechanical properties and shape memory effect, for example, with 50% strain, the SMPU-30 shows 97% of shape recovery. Thus in this study, we selected SMPU-30 and assumed that it has a characteristic structure of a shape memory polymer. Subsequently, in one way, these simulation outcomes are in good agreement with previous experimental results^29^, in key aspects and can verify the characteristics of the morphological model, namely, representative phase separation between hard and soft segments deduced by multiple measuring techniques^29^, (see Fig. 1d). In another way, it is interesting to note that, in this SMPU, the linked-spherical-phase structure of MDI are almost the same as the net-point-frame in SMP frame model which was proposed in our previous report^41^, but our simulation provides more details including a sphere diameter of around 15 nm as eight net-points connecting each other by narrow rods to form a framework to a cubic unit cell of around 30 nm in each length dimension. The PCL soft segment phase, just like a matrix, acts as the switch and is generally continuous with some concave regions to allow the MDI and BDO phases to fill in. Additionally, the phase shape of BDO is not regular and we term it as connected-spider, which has linkage in one dimension and can reinforce the MDI framework. The BDO phase apparently takes up the empty space and acts as the interphase between the PCL and MDI. It should be noted that, in existing models, there is no knowledge of BDO’s configuration in SMPUs, thus the results here may offer new insights to the functions of each block of the SMPU. Accordingly, an overall morphological architectural model shown in Fig. 4 can then be put forward and the functions of each component can be explained more specifically due to the above details being obtained.

In summary, the results of the current DPD simulation can first clearly describe the phase separation and domain details of the SMPU, which validates the morphological model of the SMPU, and secondly verifies vividly the proposition for the switch-net-point shape memory structural model proposed in our previous report^41^ by the MDI framework and PCL matrix. Thirdly, the 3D unit-cell structure, its dimensional specifics and the configuration of BDO obtained by the simulation, lead to more understanding of the SMPU structure and individual functions of each component in the shape memory effect as shown in Fig. 4c, which significantly enrich the existing models. Finally, this work can integrate the two existing two models by projected 2D morphological images and 3D architecture of the SMPU.

This simulation approach can overcome the previous limitations by providing direct evidence for the proposition of our shape memory frame model and a better understanding of the experiment results with more details such as dimension size, connection information among net-points as well as the interphase between the switch and network. Generally speaking, we obtained a unit-cell structure for shape memory polymers to show the smallest functional architecture for the first time, which can provide all the connection details and integrate all the current shape memory models and may lead to a new start for the study of mechanism of shape memory polymers. This unit-cell structure may be a general feature of some smart materials, thus this work can also inspire other scientists to find the unit cell for their concerned smart materials. It provides accurate insights of SMPU morphologies and serves as theoretical guides for smart materials design. The simulation method for polymer structure at nanoscale can be extended to many other applications where nanoscale self-assembly plays a vital role, such as photonic crystals for structural color materials.

## Methodology

It is true that the DPD method is fairly established for simulating block copolymers. Some good results have been obtained by using this method to support experimental investigations such as refs 35, 36, 37, 38, 39, 40. For standard or simple polymer structures, it is effective to get beautiful data. However, these do not mean that the DPD method has been entirely understood, fully explored, and everyone can easily become proficient to use. Instead, it is just a beginning for discovering new phenomena of smart polymers and there are a lot of new problems emerging. From our experience, the parameter design of shape memory polyurethanes for DPD simulations is an extremely difficult process, as a shape memory polyurethane normally has long soft segment block and very short hard segment block with very small extender molecules. The following is the description of our methodology for current simulation after enormous experiments.

In the DPD method, a group of atoms or a volume of lipids are modeled as soft beads, representing fluid elements rather than real particles, which is large on the atomistic scale but still macroscopically small^43^. The motion of DPD beads is assumed to be governed by Newton’s laws^43^.


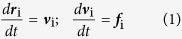


where ***r***_i_ and **ν**_i_ are the position and velocity of the *i*th particle, respectively. All the masses are normalized to 1 for simplicity, and the force acting on a particle is a sum of three pairwise contributions: a conservative force ***F***^C^, a dissipative force ***F***^D^, and a random force ***F***^R^, i.e.,


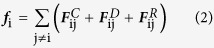


where the sum runs over all other particles within a certain cutoff radius *r*_c_. As this is the only length-scale in the system, we use the cutoff radius as our unit of length, *r*_c_ = 1. The different parts of the forces are given by:


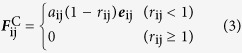



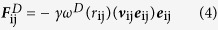



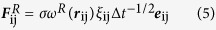


where ***r***_ij_ = ***r***_i_ − ***r***_j_, 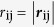, ***e***_ij_ = ***r***_ij_/*r*_ij_, ***v***_ij_ = ***v***_i_ − ***v***_j_. The parameter 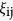 is a random number with zero mean and unit variance. The parameter ***a***_*ij*_ is a constant which describes the maximum repulsion between interacting beads. The parameters *ω*^D^ and *ω*^R^, respectively represent *r*-dependent weight functions for the dissipative and random forces, and vanish for *r* > *r*_c_ = 1. Unlike the conservative force, the weight functions *ω*^D^(*r*_ij_) and *ω*^R^(*r*_ij_) of the dissipative and random forces couple together to form a thermostat. Español and Warren have shown that there is fluctuation-dissipation theorem in the dissipative force and the random force^44^:


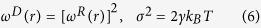


Here we choose a simple form of *ω*^D^and *ω*^R^ following Groot and Warren^43^,


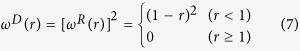


The Newtonian equation of position and velocity of particles is solved by a modified version of the velocity Verlet algorithm^45^. In the simulation, the radius of interaction, the particle mass, and the temperature were chosen as *r*_*C*_ = *m* = *k*_*B*_*T* = 1 and *σ* = 3.67, while the particle density *ρ* = 3 (taking into account the computational efficiency, *ρ* = 3 is a reasonable choice). The only parameter to be determined is the maximum repulsive force *a*_ij_, which is chosen according to the linear relation with Flory–Huggins *χ* parameters^46^:


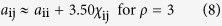


The *χ* parameter between DPD pairs of particles can be obtained from the solubility parameters using:


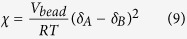


Here, *V*_bead_ is the volume of polymer segment corresponding to the particle size in the DPD, R is the molar gas constant (J/(mol∙K)), T is the temperature in Kelvin, at which simulation is performed and the parameter δ is the solubility parameter.

### Volume of DPD beads

From the introduction above of DPD theory, some parameters are needed to perform the DPD simulations. First, the volume of simulation beads should be determined to construct a mesoscopic model. Different beads representing a number of monomers are assumed to have equal volume, which is necessary to conform to the Flory–Huggins theory and the standard DPD model^47^,^48^. The same density of beads for all species is restricted to make a stronger link with experiments^49^. The volumes of the monomers are obtained by quantitative structure property relationship (QSPR) methods^50^, which are available in the synthia module in Materials Studio software^51^. Six PCL (polycaprolactone) monomers are taken as one DPD bead, so the reference volume of one bead is about 600 Å^3^, approximately equals to the volume of 3 MDI (4,4′–diphenylmethane diisocyanate) monomers, or 8 BDO (1,4–butanediol monomers), as shown in Table 1.

### Number of DPD beads in each polymer chain

**Figure 5:**
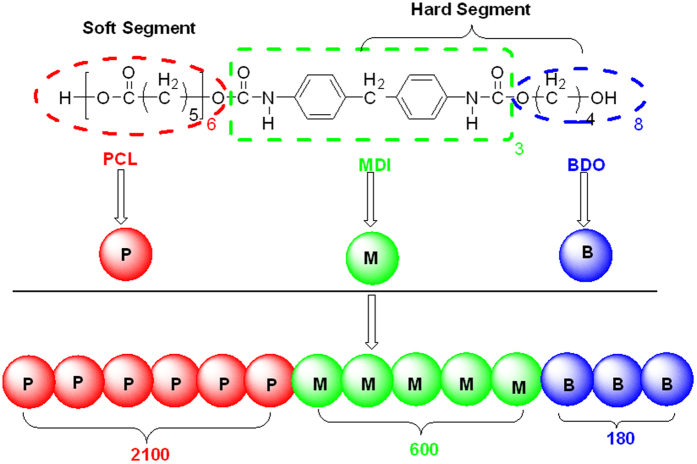
Construction of DPD beads of SMPU model.

In present study, we designed the composition of shape memory polyurethanes based on the composition ratio of our previous work^29^, as shown in Fig. 5. The soft segment PCL (polycaprolactone diols) is regarded as one type of bead, while hard segment MDI (4,4′-diphenylmethane diisocyanate) with chain extender BDO (1,4-butanediol) are two different types of beads for simulations, and the SMPU is treated as a tri-block copolymer. To ensure all the blocks have integer monomers and integer DPD beads, we designed the SM polymer with much larger system, including 2100 PCL DPD beads, 600 MDI DPD beads, and 180 BDO DPD beads.

### Interaction parameters

Solubility parameters (δ) for three components (PCL-MDI-BDO) of the system, generated using QSPR methods with Synthia^51^, are given in the Table 2.

Integrating equation (8) and equation (9), the Flory-Huggins χ parameters and maximum repulsive force *a*_ij_ can be calculated, as shown in Table 3. The pair interaction parameters provide integral measure of interaction strength.

### DPD simulations

DPD simulations of the SMPU were performed in a cell of size 30 × 30 × 30, with bead density ρ = 3, containing about 8.1 × 10^4^ DPD beads, and periodic boundary conditions were applied. Although a larger simulation box would be better to avoid finite size effects, it was too time-consuming, and it was found that, the size of simulation box does not affect the radius of the phase domains^52^. Thus this box was adopted. The finite size effects should not have been significant, since there were at least one domain in the present system for polymers of length N = 30 studied in this work. For convenience, the particle mass m, and k_B_T were all taken as unity. The time step ∆t was taken as 0.05^53^, and adjacent particles in the polymer chain interacted via a linear spring with a harmonic spring constant of 4.0, according to Groot and Liu^54^,^55^,^56^. Additionally, the friction coefficient γ was chosen as 4.5^56^. A total of 8 × 10^4^ DPD steps were carried out for a DPD simulation in this work. All the DPD simulations were performed using the Materials Studio software package^51^.

## Additional Information

**How to cite this article**: Hu, J. *et al.* Revealing the morphological architecture of a shape memory polyurethane by simulation. *Sci. Rep.* **6**, 29180; doi: 10.1038/srep29180 (2016).

## Figures and Tables

**Table 1:** Number of monomer in one DPD bead.

Monomer	Monomer volume V_m_(Å^3^)	Monomer number per DPD bead (N)	Bead volume V_bead_ (Å^3^)
PCL	103.6	6	621.6
MDI	199.3	3	598.0
BD	74.22	8	593.8

**Table 2:** Interaction parameters (δ).

Species	δ (J·cm^3^)^1/2^
PCL	17.65
MDI	27.15
BD	19.34

**Table 3:** Bead-bead interactions in DPD model.

Species	χ	a_ij_
PCL–MDI	21.61	95.66
PCL–BDO	0.5969	26.95
MDI–BDO	14.67	72.97
